# Hyperinsulinemia during in vitro oocyte maturation changes gene expression of insulin signaling in bovine Day-8 embryos

**DOI:** 10.1186/1751-0147-57-S1-O10

**Published:** 2015-09-25

**Authors:** Denise Laskowski, Hans Gustafsson, Patrice Humblot, Ylva Sjunnesson, Göran Andersson, Marc-André Sirard, Renée Båge

**Affiliations:** 1 Department of Clinical Sciences, Swedish University of Agricultural Sciences, Uppsala, Sweden; 2 Department of Animal Breeding and Genetics, Swedish University for Agricultural Sciences, Uppsala, Sweden; 3 Centre de Recherche en Biologie de la Reproduction, Department des Sciences Animales, Laval University, Québec City, Québec, Canada

## Introduction

The obesity-metabolic-syndrome-complex is a growing problem in humans as in other species and known to be associated with decreased fertility. Insulin concentrations differ from physiological levels during periods of metabolic imbalance. The dairy cow suffers from metabolic disturbances due to a stressed metabolism caused by high milk-production.

## Objective

The aim of this study was to investigate the effect of insulin during oocyte maturation on gene expression of insulin signaling in bovine Day-8 blastocysts (D8BC).

## Material and methods

Embryos (n=120) were produced in vitro according to standardized methods and divided into three maturation groups with different insulin levels (High 10 µg/ml; Low 0.1 µg/ml and Zero=control). Gene expression data of D8BC was received through microarray studies after total-RNA-extraction at the EmbryoGENE®-platform using a 2-colors-dye-swap design. Differentially expressed transcripts between control and insulin treated groups were searched by using an empirical Bayes moderated t-test in the ‘limma-package’ of R and defined as having a 1.5-fold change difference and p<0.05. Data was analyzed using IPA (http://www.ingenuity.com) to construct an overview over the insulin signaling pathway.

## Results

Both genes associated with insulin resistance (PSMD4; PSMC1; MAP2K2) and its countermeasures (ADIPOR2; INSIG1) were up-regulated by insulin treatment.

## Conclusion

As we could confirm important changes in gene expression of the early embryo, we might conclude that even short-term exposure to insulin during early development could lead to disturbances of important metabolic pathways with unknown consequences for the functions of metabolism later in life. **Funding:** The study was financed by FORMAS.

